# A qualitative exploratory interview study on birth companion support actions for women during childbirth

**DOI:** 10.1186/s12884-022-04398-4

**Published:** 2022-01-22

**Authors:** Eva Wodeya Wanyenze, Josaphat K. Byamugisha, Nazarius Mboona Tumwesigye, Patience A. Muwanguzi, Gorrette K. Nalwadda

**Affiliations:** 1 Department of Nursing, Mbarara University of Science and Technology, Mbarara, Uganda; 2 Department of Obstetrics and Gynecology, College of Health Sciences, Makerere University, Kampala, Uganda; 3 School of Public Health, College of Health Sciences, Kampala, Uganda; 4 Department of Nursing, College of Health Sciences, Makerere University, Kampala, Uganda

**Keywords:** Continuous support, Birth companion, Labor, Birth, Uganda

## Abstract

**Background:**

The World Health Organization recommends that women are supported continuously throughout labor by a companion of their choice. And, that companions have clearly designated roles and responsibilities to ensure that their presence is beneficial to both the woman and her health care providers. Presently, there is lack of strong evidence regarding specific support actions in relation to women’s needs of care. Thus, we aimed to explore birth companion support actions for women during childbirth.

**Methods:**

This was an exploratory descriptive qualitative study conducted between August 2019 and December 2019; at a referral hospital in the Eastern part of Uganda. Ten women were purposively selected: those who were admitted in early labor, expecting a normal delivery, and had fulltime birth companion. Nonparticipant direct observation and in-depth interviews were used to collect data. Latent content analysis was used.

**Results:**

Three themes were identified: “Support actions aiding a good childbirth experience”, “Support actions hindering coping with labor”, and “Women’s needs and expectations of care”. Support actions aiding a good experience described were; emotional presence, motivation, providing nourishments, messenger activities, body massage for pain relief, assisting in ambulation and coaching. Companion fearful behaviors and disrespectful care in form of unacknowledged needs and hostility from birth companions were reported to hinder coping. The women desired thoughtful communication, trust, for birth companions to anticipate their needs and recognize non perceptive phases of labor to allow them focus on themselves.

**Conclusion:**

Birth companions from this study largely supported women emotionally, and attended to their physical needs. The greater part of support actions provided were esteemed by the women. Presence of birth companion will be of benefit when individual needs of women are put into consideration. Also, more guidance for birth companions is necessary to boost their role and mitigate shortcomings of their presence during childbirth.

## Background

Birth companionship during childbirth is a vital culturally sensitive feature of respectful maternity care [1]. The labor and birth period is associated with a number of life threatening complications including; hemorrhage, sepsis, asphyxia and psychological distress [2]. The World Health Organization (WHO) recommends that every woman and her family be provided with emotional support that is sensitive to their needs. Also, that women are supported continuously throughout labor by a companion of choice [3]. Support during labor is defined as the presence of a trained professional or lay person, or family members at the bedside of a parturient woman, to coach, empathize with, give practical aid to, and inform the expectant mother about birthing [4]. Women who are continuously supported during labor are more likely to have a spontaneous vaginal birth, shorter labor, a baby with a high five-minute Apgar score and less likely to report negative ratings about their childbirth experience [5]. For implementation considerations, the WHO recommends that labor companions have clearly designated roles and responsibilities. This is to ensure that their presence is beneficial to both the woman and her health care providers [3]. Presently, women in Uganda are allowed to have a companion of choice during labor but have no orientation nor defined roles and responsibilities.

Generally, there has been progress in the maternal mortality rates in Uganda. This has been mainly attributed to skilled birth attendance with the national percentage at 74 %[6]. The maternal and neonatal health care indicators and women’s experience of care however persistently remain poor [6, 7]. Accelerating the reduction of intrapartum-related maternal, fetal, and newborn mortality and morbidity in a low-income country like Uganda will require deliberate interventions targeting the process of labor and birth [7]. Labor companionship like other non-clinical interventions has not been regarded as a priority in many settings yet it is an essential component of the experience of care [8]. Several barriers have been identified in the implementation of companion of choice at birth in resource strained hospital settings. Some of these barriers are; crowding of shared labour rooms, absence of clear communication with the companion about the role of the companion and possible interference with activities in the labour ward [9]. The absence of national implementation guidelines presents challenges for the implementation of continuous support during childbirth [8, 10]. Presently, there is lack of strong evidence regarding specific support actions in relation to women’s needs of care. It is also important that companionship takes into account the preferences and aspirations of women in labor [11]. The study findings may perhaps facilitate the development of a tailored companions’ training model to address role clarity [12].

The aim of this study therefore, was to explore birth companion support actions with reference to women’s needs of care during labor and birth. The guiding questions for this study were; ‘What support actions do birth companions presently provide for women during childbirth? What are the women’s expectations of care from birth companions?

## Methods

### Design and participants

This was an explorative descriptive qualitative study underpinned by a constructivist philosophical paradigm. In this paradigm, the researcher evaluates what is seen and said to ascertain the facts. The aim is to find the accurate state of the situation under study in the natural setting for the researcher to experience the phenomenon and construct meanings [13]. This design was preferred because it allows the target phenomenon to present itself without manipulation as it would if it were not under study for direct observation [14, 15]**.** In this study, nonparticipant direct observation of the woman and her birth companion was used to explore birth companion support actions. Additionally, in-depth interviews with the woman were used to explore her needs and expectations of care. Observation notes directed the interview session to clarify how the noted actions made them feel. Sample size determination was guided by the principle of data saturation; That is when no new information emerges from the subsequent participants [16]. Participants were purposively selected at the maternity unit by the first author (EWW). This strategy enabled deliberate choice of participants who met the inclusion criteria. These were; women who had a birth companion, in spontaneously established labor at 4 to 6 cm cervical dilatation and, expecting a vaginal delivery. To ensure that the participants were distributed relatively to the eligible population, maximum variation was employed during the sampling process to include different characteristics of women and companions. Approximately 2950 women delivered during the study period including caesarean births, women admitted in second stage, women who gave birth on their way to the hospital and complicated cases from lower health centres. Fourteen women consented to participate in the study. Four were dropped because they had an emergency cesarean section and follow up on the support actions was not possible for the normal process of childbirth.

### Setting

Participants were recruited from Mbale Regional Referral Hospital (MRRH), a government funded hospital in the Eastern part of Uganda. The sub-region is home to mainly the Gisu people that mainly practice farming with an average household size of about 4.8 people [6]. This hospital was selected because it is the main referral hospital for the region serving a diverse group of women. The maternity unit manages both complicated and uncomplicated births with about 600 births per month. In 2016, the maternal mortality rate for the region was at 535/100,000 live births compared to the national average 343/100,000 live births. Antenatal care from a skilled provider was at 97%. Of these, only 47% completed 4 antenatal visits [6].

### Data collection procedure

Data were collected between August 2019 and December 2019.

### Observation

On arrival at the maternity unit, EWW selected one woman for the day to explore full details of the support provided for the selected participant. Consent was then sought from the woman and her companion. Observation commenced from the point of admission till 1 h after birth for each participant with an average duration of 8h. Observation was focused on viewing the support actions provided by the birth companion. Careful observations were made looking out for the general sequence of events, supportive actions provided by the birth companion, actual interactions including physical, verbal, and nonverbal cues. To minimize the Hawthorne effect, the observer tried their best not interfere or manipulate with the care of the woman in labor but focused on the support actions being provided for the woman [17].

### Interview

Following birth, an in-depth interview was conducted the following day. This was conducted in the absence of her companion in the meeting room at the postnatal unit before discharge by EWW. This interview was conducted to ascertain the woman’s needs, beliefs and feelings with regards to the support care received from the birth companion. The interview questions were developed basing on literature regarding women’s needs of care during childbirth and the responsibility of the birth companion [18]. Participants were asked open-ended questions like; “please tell me about your experience of labor.”, “tell me about the things that your birth companion did for you”. In addition, the interviewer probed if the companion met her desired care”. For example; “From your description, you talk about being between life and death. What do you mean?”, “When they forced you to drink, how did that make you feel?” The interviews were audio recorded with participant’s permission and each interview lasted an average of 45 min.

### Data management and analysis

Data were analyzed simultaneously with data collection reflecting and modifying the process of data collection with the subsequent cases [19]. Latent content analysis was used; this enabled in-depth interpretation of the underlying meanings of the text and condensing data without losing its quality [20]. The audio recordings were transcribed verbatim and were then de-identified. The researcher familiarized themselves with the data. Analytical notes, thoughts, and reflections were made in this process with the aim of comprehending data collected. Data were then broken down into smaller meaning units; and each identified meaning unit was labelled with a code, as understood in relation to the objective of the study. Codes were generated inductively; these were compared with the original text to ensure that meaning had not changed [21]. Data were coded manually by EWW and PAM; GN provided oversight and confirmed the codes. GN oversaw the coding process. The codes were grouped into subcategories, categories, and then themes were formulated based on both the data and research question [22]. The analysis was deliberated among all co-authors for clarity. Narrative quotes that best described the various categories were also selected.

For rigor and trustworthiness of data, triangulation was done by combining observation and interviews, continuous observation, peer debriefing after data collection, and detailed descriptions. Furthermore, independent analysis was done by GN, and all co-authors contributed to analytical rigor and confirmed the findings. At the time of the study EWW was a midwife and lecturer in a government University. EWW, PAM and GN also had reflexive journals to account for potential biases during data collection and analysis [23].

### Findings

A total of ten women were observed and interviewed with an age range of 17–32. Majority were first time mothers all with at least primary level of education. Birth companions included mothers, aunts, sisters, and friends. All were married except for one (see Table 1).

**Table 1:** Participant’s characteristics

No.	Age	Parity	Marital status	Highest level of Education	Birth companion
1	20	2	Married	Secondary	Husband’s mother
2	21	1	Married	Tertiary	Aunt
3	17	1	Single	Primary	Aunt
4	26	3	Married	Secondary	Husband’s sister
5	18	1	Married	Secondary	Mother
6	32	3	Married	Tertiary	Friend
7	23	1	Married	Secondary	Mother
8	29	2	Married	Tertiary	Sister
9	29	3	Married	Secondary	Sister
10	19	1	Married	Secondary	Friend

Findings are presented under three major themes, “Support actions aiding a good childbirth experience”, “Support actions hindering coping with labor”, and “Women’s needs and expectations of care”. Observation notes mostly contributed to the physical actions established; while the interviews contributed mostly to ascertaining the emotional actions and what women desired from their birth companions.

### Support actions aiding a good experience of childbirth

This theme emerged from three categories; “emotional helpfulness”, “physical assistance”, and “instruction and advocacy”. The development of theme 1 is shown in Table 2.

**Table 2:** Development of theme 1: Support actions aiding a good experience of labor

Meaning unit (from observation notes and interviews)	Condensed meaning unit	Code	Category
…for somebody to sacrifice their time, yet she had somewhere else to go. she is there in that situation with you.	Selflessness, Emotionally partaking in her labor	Emotional presence	Emotional helpfulness
…but they stayed with me, I was abusing them but they helped me.	Unconditional emotional support		
she was there and was willing to see what was going on. She was there even though I tugged her tightly she was there she stood there for me.	Submissive presence		
being present for me to know that I have my people. They were there	Available for emotional support		
… the pains are too much” she is groaning with pain, “I know, but you have to be strong God will help you”.	Considerate of her pain		
The courage she gave me was that, even if I pulled her, she was near me and told me, “Be strong God will help you”	Encouragement	Motivation	
Every time I felt hopeless, she encouraged me to be strong. She told me to be strong. She told me, “I came here for you, you have suffered all this time, you can still be strong. I was feeling so much pain. But I was strong like a woman.	Boosted confidence in a seemingly hopeless situation		
...companion has offered hot tea, “drink”, …she then leaves the room and comes back with a drink and something to eat …She gave me something to eat	Provide nourishment to meet the demands of labor	Providing nourishment	Physical assistance
…the midwife cleans her up the companion leaves the room to bring more clothes. The midwife asks for cotton; the companion looks for the cotton in the bag and hands to the midwife …Second Companion brings in required items for the midwife ...she was there, when she was asked by the midwife to bring something, she brought the things she was sent for. …when you are in labor, she gets for you the things you need.	Co-player with the midwife in providing care	Messenger activities	
...…. the woman bends on the bed. Companion holds her back and presses firmly on the back.... The contraction is over, she tells her to get off the floor, she holds her and helps her to get up. …She made me strong, I felt a lot of pain and she kept pressing my back…the pain was too much, I told her, to press my back, she did the things that I wanted. Even if the pain did not go away, it felt good. I was feeling a lot of pain…I was not aware what was going on. I held her hand, she held my hand. I held her hand tight	Efforts to alleviate pain. Touch and massage communicate care to the labouring woman	Non pharmacological pain relief	
…and held me up and walked with me she held my back. …I had lost hope they told me to walk. They supported me to walk slowly. I went to walk up and about slowly. But I was with my mother, my mother was walking with me. They told me to walk, I walked the whole field, when I would finish walking, I would come back and take tea.	Companion inspired her to walk	Assisting in Ambulation	
…she corrected me when I made mistakes and she advised me to follow the instructions of the midwife. The midwife checks on her, instructs her how to push with the following contractions. The companion is holding her telling her to be strong and to push the baby.	Birth companion augmenting instruction Task shifting	Guiding	Instruction and advocacy

### Emotional helpfulness

Women in this study reported that birth companions boosted their confidence in a seemingly hopeless situation. A psychologically safe environment with continuity of emotional support is significant for a positive childbirth experience. Confidence during labor creates a sense of control and women may perceive their labor as less painful. Birth companions unconditionally stayed with the women and partook in the events of labor submissively.

*… Companion comes in, she tells her to be strong and that she will deliver the baby successfully … Companion smiles, and says “you are getting there, you will be alright”.*

(Observation notes: Second child, 20 - Husband’s mother)

They recounted that birth companions were willingly present and ready to do what was required of them. This sacrifice and presence were comforting and gave the women a sense of security in an unfamiliar environment.

*… when the midwife called her to come, she came quickly and she was there a lot and was willing to see what was going on … She also got worried … she started praying.*

(Third child, 29 - Sister)

### Physical assistance

Birth companions support actions physically assisting women found were; ‘providing nourishment’, ‘messenger activities’, ‘body massage for pain relief’ and ‘assistance to walk’. Providing tea was the main form of care among all the birth companions. Companions were seen providing warm drinks and light foods to the women. They associated taking hot tea with hastening the labor process and pain relief. The process of labor involves extensive uterine muscle contractions thus requires a considerable number of calories. Frequent sips of sweetened tea help to provide energy and improves hydration necessary for labor.

*The pain was too much, my whole body was shaking, I did not have the energy ... I tried to push but could not. When they gave me more tea, I gained the energy to push.*

(First child, 23 - Sister)

Birth Companions further performed messenger activities including purchasing and making available supplies like cotton, gauze, mackintosh*,* gloves, disinfectant, and clothes. Some of them came with pre-packed supplies while others kept running out to buy from the nearby shops.

*when you are in labor, she goes to get for you the things you need, she comes to check on you, does what the midwives says until the end of the delivery … She does a big job.*

(Third child, 29 - Sister)

Some form of pain relief during labor is necessary to enhance a good birth experience. Birth companions in this study attempted to relieve pain through bodily touch. Some companions were seen rubbing the laboring woman’s back, while others simply hugged them and told them to be strong. The women said that while the back rub did not take away the pain, it was comforting. The birth companion was their nest to relinquish their pain. This action communicates care to the woman and enables the companion to partake of the pain and promotes bonding and trust between the woman and her companion.

*The companion is sitting on a stool in the room, she is looking at the woman, she asks her if the pains are coming frequently … she then rubs her back.*

(Observation notes: First child, 21 - Aunt)

Birth companions furthermore walked with the women in labor. Companions encouraged them to walk even when they women did not feel like walking. The women said the walking helped them even though they were tired at certain moments. Mobility and an upright position during labor are encouraged to promote the descent of the baby and pain relief.

*They said, “get up and walk, if you sit even the baby will not come out”. So, they encouraged me, they told me to walk. I had lost hope, they told me to walk. They supported me to walk in the compound slowly.*

(First child, 23 - Mother)

### Instruction and advocacy

Companions linked the midwife and the woman by augmenting the midwives’ instructions. They supported the women to follow through the instructions of the midwife. The presence of the birth companion promotes effective communication necessary for good quality care during childbirth.

*The midwife re assesses the woman. Checks on the fetal heart. The woman says she is tired, the midwife advises her to have more tea. The companion tells her to get off the bed to take some tea,*

(Observation notes: Second child, 29-Sister)

They encouraged the women to trust the midwives. Some birth companions were heard sharing their birthing experiences with the woman and lessons learned from their own birthing experiences on how strong and brave they were.

*...She said, “even if the contractions are so bad, don’t do anything until the midwife tells you what to do;”. if you feel like passing stool, that is the baby, do not scream … the contraction will guide you when to push.”*

(First baby, 21- Aunt)

### Support actions hindering coping with labor”

This theme emerged from two categories, “Fearful actions” and “disrespectful care from companions” as illustrated in Table 3.

**Table 3:** An illustration of the formation of theme 2: Support actions hindering coping with labor

Meaning unit (observation notes and interviews)	Condensed meaning unit (Interpretation of the underlying meaning)	Code	Category
When I looked at her, she was crying, every time I saw her cry, I also began crying... …but for her she was scared…for her she stayed outside. She did not even enter. *…*Companion comes back in the room she seems too scared, she is sweating, she goes back outside*.*	Companion anguish aggravating woman’s distress Discouraging non-verbal communication	Distressed companion	Companion fearful behaviors
companion is pacing up and about. She is putting her arms on the head. …The companion looks very disappointed she is sweating profusely. … Once we reached here, she ran away	Nonverbal cues of giving up Over whelmed companion	Hopelessness	
The two women look furious, “push the baby,” one slaps the woman’s thighs … they slapped me in the mouth…. Since yesterday they beat me	Antagonistic means of dealing with dissatisfaction of woman’s efforts.	Hostile actions	Disrespectful care from companions
…. I wanted to take water…. since morning they refused me to take water.	Deprived of basic needs	Unacknowledged needs	

### Fearful behaviors

Some birth companions were overwhelmed with the labor process and reacted to the women’s anguishes with distress. Some companions were seen crying, pacing constantly and holding their hands above their heads. This amplified the women’s assessment of the situation yielding more anxiety making it difficult for them to cope with the pain.

*she had also given up … she was crying, I felt like I was increasing her tension. I was worried that her blood pressure would rise. So, I was demotivated.*

(First child, 23 - Mother)

### Disrespectful care from companions

During the critical moment of delivery, some companions felt the need to intervene; Birth companions were seen hitting or slapping the women; two women reported to having been slapped. Other interventions included holding their mouths, restraining their legs on the bed, and lifting the woman’s head off the bed to prevent her from lying flat on the bed. This was out of desperateness of the birth companions to have a good outcome irrespective of what the women wanted. Whereas some women found this disrespectful and unjust, one participant thought that it was for their own good after all. They did not mind about the hostility rather they were happy that it all worked out for their own good.

*I knew that she was trying to help me because I had pushed, and the baby’s head was already out, and the rest of the body was inside, so she slapped me, she was trying to help me.*

(First child, 17 - Aunt)

Some women were denied drinks and food by their companions for the reason that it would affect the progress of the woman’s labor.

### Women’s needs of care

This theme emerged from these categories; “thoughtful communication”, “recognize unperceptive phases”, “anticipate needs” and “trust” as illustrated in Table 4.

**Table 4:** An illustration of the development of theme three, “women’s needs and expectations of care”

Meaning unit	Condensed meaning unit (Interpretation of the underlying meaning)	Code	Category
You really need a lot of encouragement…	Build confidence	Reassurance	Thoughtful communication
you don’t need anyone to bark at you, you just feel irritated, they forced me, “take tea”. I felt like everyone should go away from my side… If someone forces you to do something you only feel negative.	Shouting makes her feel less in control of the situation.	Insensitiveness	
I feel polite language is important. It is for this reason that I remained with my cousin instead of my mother. She had started to shout at me. She said, “don’t make for us noise, you keep quiet”. I felt so bad that my mother was talking to me like that.	Demonstrate respect in all support actions	Respect	
	Avoid orders	Polite language	
there those who you come with who shout and beat you. “You have not done this, push very fast”. There is no need for shouting.			
If she is telling you something, it should not be said forcefully. Even me I know I am supposed to eat so that I get energy.			
...when you are in labor, you lose your senses. The person who is attending to you has to be someone who is soft hearted.	Recognize moments when she should be “left alone”	In a different place.	Recognize non-perceptive phases of labor
She says, “drink tea… do this”, you are feeling bad… struggling with contractions... she says “drink tea”, really? no! She should first leave you to relax. It is the pain that makes you rude.	Moments when external perceptions are shutting off.		
I wanted so much for a person to be around but there was no one. I held the bed tight and pushed the baby	Empathize, fore see what someone in that situation would want	Could not understand what I wanted	Anticipate needs
…I wanted her to hold my back			
. I wanted someone to be around, to hold me and touch my back. The back felt like it was going to break into half.			
…the pain was too much I wanted someone to hold my hand and walk with me			
… it needs someone you trust. Like you take them as your friend. You need your close friend so that you can do anything and whatever happens in the hospital stays there. They will not take it outside. Saying, “while in the hospital, she behaved like this”. So, it needs somebody you can trust	Freedom to being	Needs someone you trust	Trustworthiness

### Thoughtful communication

Effective communication is key in respectful maternity care, some participants in this study felt that companions were using militaristic methods to achieve what they thought was good for the woman. The women expressed the need to be constantly reassured. This boosted their confidence and made them feel that they were in control of the situation. The women also needed their birth companions to demonstrate respect in all their support actions. One participant stated,

*I don’t want someone to shout at me. It is demotivating, at least if someone is there to encourage you. The person you have brought to help you should not be the one insulting you...*

(First baby, 23 - Mother)

### Recognize non-perceptive phases of labor

Furthermore, women needed their companions to recognize moments when they needed to be “left alone”, moments when external perceptions were shut off. They reported being lost in their own world not apprehending what the birth companion was instructing them to do.

*...At that moment, you feel like the life you have is not yours, you are in a different place. So, you may even do something and even the person who has brought you feels offended.*

(Third child, 32 - Friend)

### Anticipate needs

Care given during labor ought to be responsive to women’s needs. Some women in this study expected their companions to know what they needed without voicing them. They desired that their companions anticipate their needs because they were already dealing with so much pain to communicate what they wanted.

*… they told me to get off the floor, they told me it was dirty, they told me to climb the bed but I could not, I wanted their support, I wanted someone to hold me and help me climb the bed. They could not understand what I wanted... I felt so bad.*

(Second child, 29 - Sister)

Additionally, trustworthiness was an important attribute women needed from their companions. This enabled her freedom to do and say anything during her birth. Choice of the companion is also an important factor for a good experience of care during childbirth.

## Discussion

In this study, we explored birth companion support actions in relation to women’s needs of care to enlighten the presence of companions of choice at birth. Three themes were identified, “Support actions aiding a good childbirth experience”, “Support actions hindering coping with labor”, and “Women’s needs and expectations of care”. According to the findings of this study, birth companions were mainly emotionally helpful and attended to women’s physical needs. Some actions however hindered women’s coping with labor.

The current study found that the presence of a known birth companion for emotional support was a key supportive care action, similar findings are also reported in studies from South Africa, US, and Thailand [24–26]. This finding may perhaps contribute to existing evidence to aid implementation in similar settings. The need for the presence of the companion in the current study however was not absolute. Some women needed their companions to recognize moments when they wanted to be left alone. This finding is similar to results from a study on the emotional journey of labor; where women illustrated this moment to moving into a ‘zone’ of timelessness and letting go of control [27]. This result may perhaps suggest that birth companions recognize such moments to allow the woman’s innate need to focus on herself with each contraction. Additionally, Afulani et al. (2018) found that women’s desires for the presence of a companion varied across the stages of labor and delivery, with most desiring companionship during the first stage of labor but not at the time of delivery [12]. Presence of a companion is key; however, these findings might possibly inform the need to assess individual needs of women during childbirth.

Birth companions further physically assisted women mainly by providing nourishment. They mostly gave tea and light food. Some companions however, denied the woman drinks. The later finding is similar to findings from a study in Malawi [28]. The WHO model for intrapartum care recommends oral fluids and food intake, and adoption of mobility for a positive birth experience [29]. Further guidance for birth companions regarding fluids and food intake could be beneficial for companions. Birth companions in the current study further made available supplies needed for labor like cotton, gloves, towels, and clothes. Similar findings are also reported in studies from India and Nigeria [30, 31]. Birth companions not only provided companionship, but also took up the active role of monitoring labor and purchased drugs and items around the time of delivery [31]. Due to the frequent shortage of essential supplies and large numbers of patients in Uganda, women are asked to come with some basics like cotton, surgical gloves and gauze. This action is possibly of assistance in meeting this shortage.

Companions further linked the midwife and the woman by augmenting the midwives’ instructions. This finding is similar to a study that found that the support person mostly imitated the professional caregiver’s instructions [32]. Women from the current study however expressed their dissatisfaction with the manner of instruction. They desired respect from companions while giving these directives. This is similar to findings from an Australian study [18]. Alden et al. (2013) also suggest that the way in which support members approach labor is related to the manner in which they have been socialized to the birthing process [33]. This finding could suggest the need to explore cultural perspectives to harmoniously sequester practices that antagonize the efforts of a good birthing experience. This support action with coaching could be utilized greatly in settings with large numbers of women in labor with a few midwives.

Efforts to relieve pain was also an important birth companion support action for women in this study. Non-pharmacological methods may not lessen labor pain but can facilitate bonding between women and birth supporters [34]. Konlan et al. (2021) in a study in Ghana also found that women used various strategies in pain management, including shouting, screaming and wailing. Moreover the presence of the woman’s significant other during labor was seen to improve the women’s pain bearing ability [35]. Pain management during labor is not routinely offered in Uganda. This support action could be exploited in low resource setting by providing more direction for birth companions on the different non pharmacological techniques of relieving pain during labor and birth.

Nonetheless, some birth companion’s actions hindered women’s coping with labor. The Lazarus and Folkman’s Theory of stress, postulates that a person’s ability to cope is related to their appraisal of the stressor [36]. Seeing a birth companion anxious and distressed further distressed the women. This finding could suggest inclusion of birth companions in the woman’s antenatal care and admission process of labor to emotionally prepare them for their role.

Aggression and physical violence were also reported in this study. Some women found this practice belittling and disrespectful, while one thought it was to their advantage. Several other studies on mistreatment of women in labor have mostly focused on the health care providers [37, 38]. In these studies, some women found it disrespectful while others thought such treatment was part of the care that the providers were meant to offer [39–41]. It is perhaps necessary that there is further regulation of health care providers on maltreatment of women during labor so that they can boldly discourage the practice among birth companions.

Findings from our study highlighted birth companion support actions and women’s needs of care. Findings from this study may be of benefit in informing the implementation of the presence of a birth companion of choice at birth in similar low resource settings.

### Strengths and limitations of the study

The triangulation in data collection lessened researcher bias and assumptions that could have influenced the results of the study. The interviews accounted for the women’s thoughts and feelings versus what was observed. These findings contribute to an understanding of what women want during labor versus the support care actions offered by the person that is with them during labor. Study findings may have been affected by personal social desirability of the observer. This was mitigated by a reflexive journal to bracket personal experiences and expectations of the primary observer. Given the sample population generalization of these findings is limited to settings of a similar context.

## Conclusion

The study of birth companion support actions for women in labor showed that the presence of birth companions during childbirth is key for a positive childbirth experience. Birth companions mainly supported women emotionally, and attended to their physical needs. Some birth companion actions however hindered coping with the labor in this study. With the increasing emphasis on experience of care, presence of birth companions ought to be fostered. Implementation will be of benefit when individual needs of women are put into consideration. Furthermore, extra guidance for birth companions is necessary early in labor; to enhance their role in the birthing process and to mitigate their shortcomings.

## Data Availability

The data that support the findings of this study are available from the first and corresponding author, Eva Wodeya Wanyenze.
